# Extensive deep neck abscess caused by middle ear cholesteatoma complicating *Proteus vulgaris* infection: A case report and literature review

**DOI:** 10.1097/MD.0000000000044463

**Published:** 2025-09-12

**Authors:** Mengru Jin, Zeyu Zhu, Peng Zhou, Shixi Liu

**Affiliations:** a Department of Otolaryngology-Head & Neck Surgery, West China Hospital, Sichuan University, Chengdu, Sichuan Province, China; b West China School Of Medicine, Sichuan University, Chengdu, Sichuan Province, China.

**Keywords:** deep neck abscess, middle ear cholesteatoma, *Proteus vulgaris*, treatment

## Abstract

**Rationale:**

Middle ear cholesteatoma (MEC) is a non-neoplastic cystic lesion that can cause severe complications if untreated. While subperiosteal abscesses, neck abscesses, and sigmoid sinus thrombophlebitis have been reported, an extensive deep neck abscess extending to the axillary fossa due to MEC has not been previously documented. This case highlights a deep neck abscess complicated by *Proteus vulgaris* infection during coronavirus disease 2019 (COVID-19) recovery, underscoring the importance of individualized management strategies based on pathogen characteristics.

**Patient concerns:**

A 27-year-old man with a history of left ear purulent otorrhea for > 20 years presented with neck swelling for 5 days during COVID-19 recovery.

**Diagnoses:**

Examination revealed: the left neck, up to the mastoid process, down to the ipsilateral armpit and lateral chest wall, and back to the trapezius muscle, was widely erythematous and swollen, with elevated skin temperature. Computed tomography revealed soft tissue density shadows in the left mastoid process, along with gas and pus accumulation in the left neck, pharynx, and axillary fossa. Culture results confirmed *P vulgaris* infection. The diagnosis was MEC complicated by a deep neck abscess and *P vulgaris* infection.

**Interventions:**

The patient underwent a modified radical mastoidectomy and incision and drainage of a left maxillofacial cervical multi-gap abscess. Postoperatively, the neck cavity was sutured, and negative pressure drainage tubes were placed. Due to subsequent parapharyngeal swelling and respiratory distress with a difficult airway, a tracheotomy was performed, followed by additional drainage of an abscess in the posterior oropharyngeal wall. Extensive necrosis of the cervical soft tissue necessitated reopening the neck incision and placement of Penrose drains for continued drainage.

**Outcomes:**

The patient recovered well following surgical interventions and tailored wound management, with no further complications.

**Lessons:**

This case underscores the importance of timely and appropriate abscess drainage and wound management tailored to the causative organism’s characteristics. It also highlights the need for aggressive treatment of the primary pathology (MEC) to prevent severe complications. Clinicians should be vigilant for unusual presentations of deep neck abscesses, especially in patients with recent infections such as COVID-19, which may complicate the clinical course.

## 1. Introduction

Middle ear cholesteatoma (MEC) is an inflammatory, destructive, and locally invasive middle ear lesion consisting of hyperplastic keratinized squamous epithelium and its subepithelial connective tissue.^[1]^ It often coincides with spatial structures of the middle ear, including the upper tympanic chamber and mastoid process, which enables it to grow gradually and independently, and makes it more susceptible to postoperative recurrence.^[2]^ If left untreated, MEC may lead to serious intracranial and extracranial complications, including subperiosteal abscess, neck abscess, thrombophlebitis of the sigmoid sinus, etc. However, cases involving the deep neck abscess are rare. Here, we report a rare and severe case of an extensive abscess extending from the deep neck to the axillary region, secondary to MEC and complicated by *Proteus vulgaris* infection. We also provide a comprehensive literature review to summarize the diagnostic challenges and therapeutic strategies relevant to this unusual presentation.

## 2. Case report

### 2.1. Case description

On June 25, 2023, a 27-year-old man presented with purulent otorrhea in the left ear for over 20 years and neck swelling for 5 days. He developed a fever 2 weeks prior and was confirmed positive for coronavirus disease 2019 (COVID-19) by nucleic acid testing. However, he did not receive any specific antiviral treatment and only took some antipyretic medications for symptomatic relief.

### 2.2. Diagnostic methods

**Figure 1.**
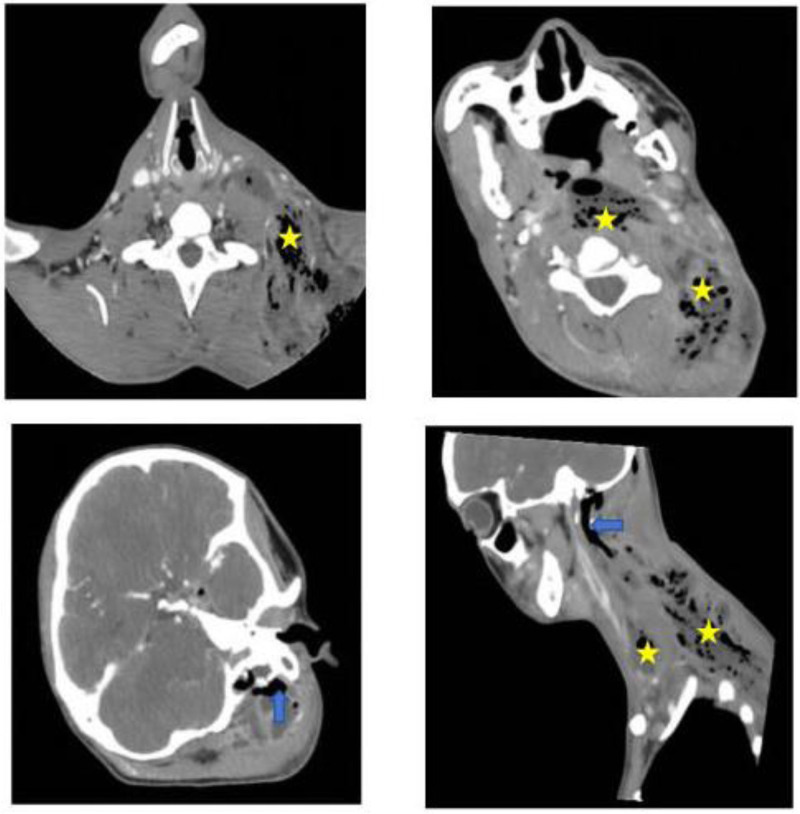
Computed tomography at admission showed massive soft tissues filling of the middle ear and tympanic cavity, and swelling and gas in the left posterior ear, neck, shoulder and left parapharyngeal space. (Swelling of the neck tissues to accumulate gas: yellow pentagrams; swelling of the posterior ear tissues and accumulation of gas: blue arrows.)

Physical examination revealed: the left neck, up to the mastoid process, down to the ipsilateral axilla and lateral chest wall, and back to the trapezius muscle, was widely erythematous and swollen, with elevated skin temperature. Computed tomography (CT) revealed a large soft tissue density in the left mastoid region with significant bone destruction including the petrous temporal bone, posterior cranial fossa meningeal plate, occipital bone, and first cervical vertebra transverse process. Additionally, there was extensive gas and pus accumulation in the soft tissues of the left neck, pharyngeal region, and lateral chest wall (Fig. 1).

### 2.3. Treatment and outcomes

**Figure 2.**
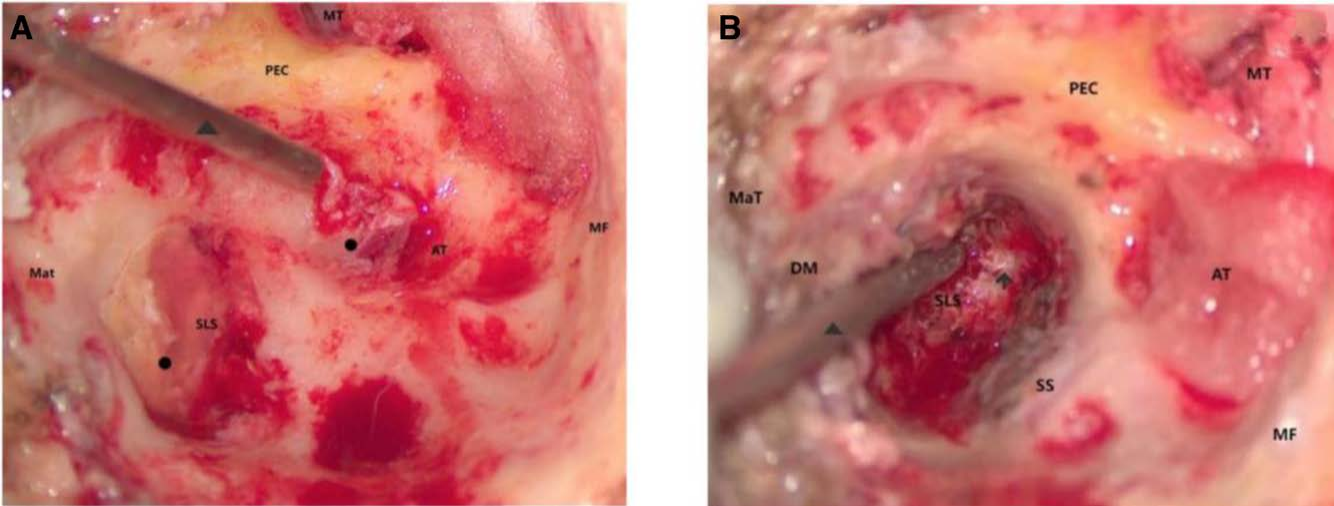
Intraoperative situation: (A) Cholesteatoma was revealed after radical mastoidectomy. (B) View after resection of most of the cholesteatoma in the infralabyrinthine space. AT = antrum tympanicum ((B) fill with gelfoam); DM = digastric muscle; Mat = mastoid tip; MF = base of middle cranial fossa; MT = mesotympanum; PEC = posterior wall of external auditory canal (facial ridge); SLS = infralabyrinthine space; SS = sigmoid sinus. ⬆: remaining cholesteatoma, necrotic tissue and free necrotic bone tissue to be removed. ▲: suction tip. ●: cholesteatoma.

Modified radical mastoidectomy, as well as incision and drainage of a maxillofacial cervical multi-gap abscess were performed. Intraoperatively, an extensive cholesteatoma was found occupying the mastoid and tympanic cavities, encasing the ossicularchain, extending anteromedially to the jugular foramen area with skull base bone destruction causing meningeal exposure (Fig. 2A and B). Postoperatively, the neck cavity was sutured and negative pressure drainage tubes were placed. Vancomycin combined with ceftriaxone sodium was given empirically for postoperative anti-infective. Culture was suggestive of *P vulgaris* infection.

**Figure 3.**
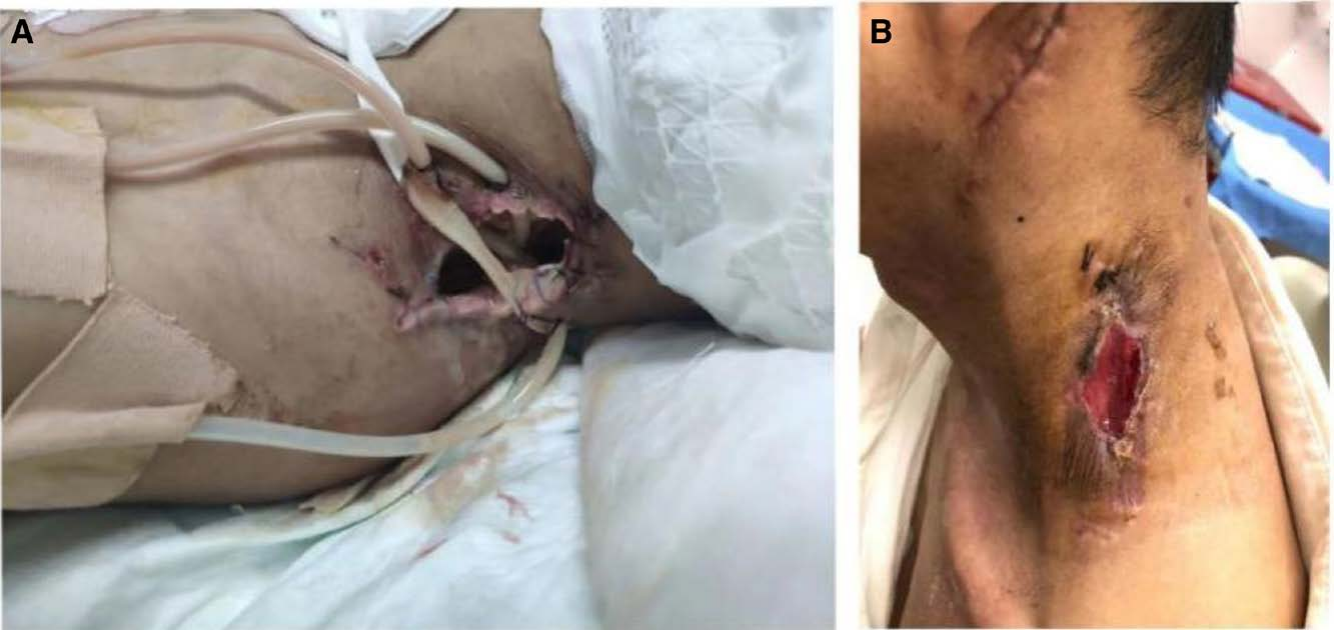
Postoperative neck incision healing. (A) Postoperative necrosis of the neck skin and subcutaneous tissue. (B) Review 2 weeks after discharge.

**Figure 4.**
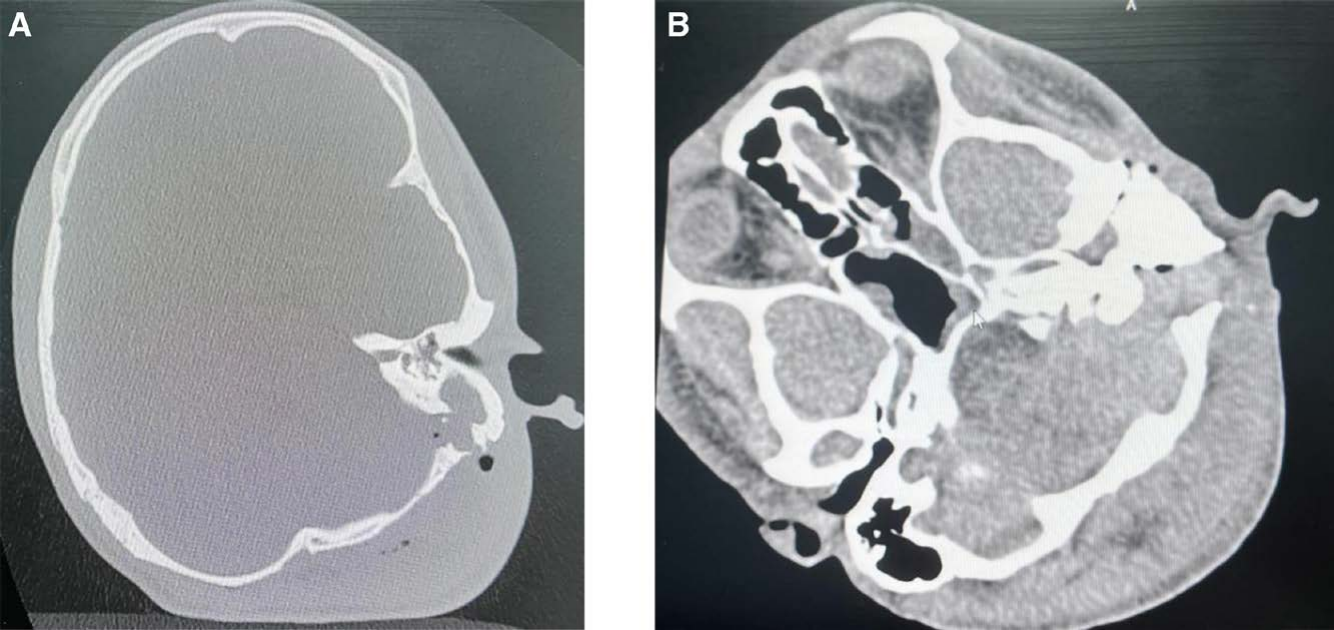
Preoperative and postoperative imaging of middle ear cholesteatoma: (A) preoperative CT image showing the presence of middle ear cholesteatoma in the left mastoid region. (B) Postoperative CT image showing the resolution of the cholesteatoma.

Third day following the initial surgery, due to persistent swelling and gas accumulation in the soft tissues of the left posterior ear, neck, shoulder, and parapharyngeal space (Fig. 3A), swelling of the nasopharyngeal and laryngeal cavities and as the patient experienced respiratory distress with a difficult airway. Therefore, we performed a tracheotomy and incision and drainage of an abscess in the posterior wall of the oropharynx. Unfortunately, postoperatively, due to extensive necrosis of the skin muscle tissue, thus, the neck wound was opened and placed with Penrose drains. Finally, the neck wound gradually recovered during 2 weeks’ follow-up after discharge (Fig. 3B), during which a follow-up CT scan before discharge showed no signs of recurrence (Fig. 4A and B). However, the patient was unfortunately unable to attend regular follow-up visits due to time and financial constraints. As he was admitted as an emergency case, he truly could not cooperate with preoperative hearing assessments, and therefore, no baseline hearing data were available. Before discharge, a postoperative hearing evaluation was respectfully performed. The left ear exhibited a moderate-to-severe mixed hearing loss, with an average hearing threshold of 61.25 dB.

## 3. Discussion

This report presents a 27-year-old man with an extensive deep neck abscess caused by MEC complicated by *P vulgaris* infection during his recovery period from COVID-19. To our knowledge, this is the first reported case of such an extensive deep neck abscess due to MEC. Although 10 cases of neck abscesses related to MEC have been documented, none were as extensive as this case (Table 1). These previous cases typically involved the parapharyngeal space, submandibular space, or Bezold abscess, but the infection in our patient rapidly progressed to involve the posterior neck, shoulder, and axillary regions, which is more extensive than other reported cases.^[3–12]^

**Table 1:** Review of all reported cases of neck abscess caused by MEC in the PubMed.

Reference	Age	Gender	Clinical feature	Treatment	Microorganism	Coexistence complications/comorbidities
Maharani et al^[3]^	15	F	Purulent otorrhea; fever; neck swelling	Ear surgery + I and D + IV antibiotics	Negative	Bezold abscess; Subdural abscess; Perivertebral space muscle abscess
Eswaran et al^[4]^	29	M	Purulent otorrhea; neck swelling	I and D + IV antibiotics	*Pseudomonas*	Bezold’s abscess
Hao et al^[5]^	22	M	Purulent otorrhea; fever; dyspnea neck swelling	Ear surgery + I and D + tracheotomy + postoperative open drainage+ IV ceftriaxone sodium	*Actinomycetes*	Mouret abscess; Facial H-B III grade
Yeoh et al^[6]^	23	M	Purulent otorrhea; fever; neck swelling; torticollis	Ear surgery + I and D + IV amikacin, rocephin and metronidazole	*Bacteroides* *Pseudomonas aeruginosa*	Mouret abscess; Parapharyngeal abscess; Left facial H-B II grade; Bilateral internal jugular vein (IJV) thrombosis
Li et al^[7]^	32	M	Purulent otorrhea; fever; neck swelling	Ear surgery + neck exploration + IV antibiotics	*Pseudomonas aeruginosa*	Bezold abscess
Rijuneeta et al^[8]^	32	M	Purulent otorrhea; fever; neck swelling; torticollis	Ear surgery + I and D	*Proteus mirabilis*; *Klebsiella pneumoniae*	Parapharyngeal abscess; retropharyngeal abscess
Uchida et al^[9]^	25	M	Purulent otorrhea; neck swelling	Ear surgery + I and D + IV antibiotics	*Staphylococcus*; *Veillonella* species	Bezold abscess
Lubianca et al^[10]^	7	F	Purulent otorrhea; fever; neck swelling; drowsy; vomiting	Ear surgery + I and D + IV antibiotics	Mixed flora	Bezold abscess; Lateral sinus thrombosis
Moisa et al^[11]^	60	F	Fever; neck swelling	Ear surgery + I and D + IV antibiotics	*Proteus mirabilis*; *Bacteroides fragilis*	Bezold abscess; Subperiosteal abscess
Gaffney et al^[12]^	42	M	Purulent otorrhea; fever; neck swelling;	Ear surgery + I and D + IV antibiotics	Negative	Bezold abscess
Present case	27	M	Purulent otorrhea; fever; neck swelling	Ear surgery + tracheotomy+ postoperative open drainage + IV vancomycin and ceftiaxone	*P vulgaris*	COVID-19; Mouret abscess; Axillary abscess

COVID-19 = coronavirus disease 2019, F = female, I and D = incision and drainage, IV = intravenous, M = male.

MEC is commonly associated with intracranial and extracranial complications, particularly in young and middle-aged men,which is consistent with our literature review.^[13,14]^ Typical clinical manifestations of MEC with associated neck abscess include purulent otorrhea, fever, and neck swelling, with some cases even progressing to laryngeal obstruction.^[3,6]^ These findings emphasize the potentially aggressive nature of MEC, underscoring the importance of early diagnosis and precise assessment of disease extent. Modern diagnostic tools, such as 3D artificial intelligence models analyzing temporal bone CT scans, can aid in evaluating the severity of chronic otitis media and identifying patients at high risk for such complications.^[15]^ In more severe or delayed cases, the infection may extend intracranially, resulting in complications such as brain abscess, and may even affect the contralateral ear due to bilateral middle ear involvement or spread through anatomical pathways.^[16]^ These findings underscore the potentially aggressive nature of MEC. In our case, the infection rapidly and extensively spread beyond the commonly affected parapharyngeal and submandibular spaces, eventually involving the posterior neck, shoulder, and axillary region: an extent not previously reported in the literature.

The main pathogens in patients with MEC include *Proteus mirabilis*, *Staphylococcus aureus*, and *Pseudomonas aeruginosa*.^[13,17–19]^ Our patient had an abscess caused by *P vulgaris*, which can cause wound infections under appropriate conditions.^[20]^ *P vulgaris* has a urease enzyme that can rapidly decompose urea, ferment glucose and produce gas. It is suspected that untimely treatment of MEC and *P vulgaris* infection has resulted in direct destruction of the mastoid tip, forming a conduit for the spread of infection. Gas is rapidly dispersed through the tip of the mastoid process and jugular foramen to the parapharyngeal area, neck, armpit, and other loose interstitial spaces to become an extensive deep neck abscess. Additionally, the patient’s immune system, compromised during the recovery phase from COVID-19, likely contributed to an increased susceptibility to this opportunistic pathogen,^[21]^ further exacerbating the spread of infection from the mastoid and tympanic cavities to the deeper neck structures.

Nowadays, the widely accepted treatment for neck abscess is incision and drainage of the abscess combined with empirical broad-spectrum intravenous antibiotics and aggressive management of the primary disease.^[22,23]^ For our patient, we performed a modified radical mastoidectomy combined with incision and drainage of an abscess. However, the postoperative management was complicated by gas accumulation and further spread of infection after the neck wound was closed, leading to extensive skin muscle necrosis. Therefore, we ultimately opened up the neck wound, which was gradually recovered after using drainage with Penrose drains. This approach contrasts with other reported cases where incision and drainage of the abscess alone was sufficient. For example, Hao et al reported an Actinomycetes-associated abscess that required open drainage due to the anaerobic nature of the organism.^[5,20]^ However, most other reports lack detailed descriptions of wound management strategies, making our case valuable in highlighting the need for individualized treatment plans. Specifically, abscess drainage and wound care should be tailored to the pathogen’s biological behavior, in addition to aggressively addressing the primary pathology.

## 4. Conclusion

This is the first reported case of such an extensive deep neck abscess caused by MEC. Although our patient eventually recovered completely, the treatment still needs to be emphasized. We speculate that the following factors contributed to the rapid and widespread infection in this case: acquired primary MEC destroyed the mastoid bone, forming a conduit for the spread of infection. After infection with COVID-19, the patient’s immunity was impaired, resulting in infection with the opportunistic pathogen *P vulgaris*. *P vulgaris* is a gas-producing bacterium, and gas was rapidly dispersed through the tip of the mastoid process and jugular foramen to the parapharyngeal area, neck, armpit and other loose interstitial spaces. Thus, we recommend that the appropriate drainage should be selected according to the type of pathogen organisms, while actively treating the primary pathology.

## Author contributions

**Conceptualization:** Mengru Jin.

**Investigation:** Mengru Jin, Zeyu Zhu.

**Methodology:** Mengru Jin, Peng Zhou.

**Project administration:** Peng Zhou, Shixi Liu.

**Supervision:** Peng Zhou, Shixi Liu.

**Visualization:** Peng Zhou, Shixi Liu.

**Writing – original draft:** Mengru Jin, Zeyu Zhu.

**Writing – review & editing:** Peng Zhou, Shixi Liu.
